# Osteoporosis prophylaxis in patients receiving chronic glucocorticoid therapy

**DOI:** 10.5144/0256-4947.2009.215

**Published:** 2009

**Authors:** Mir Sadat-Ali, Abdulmohsen H. AlElq, Badar A. Alshafei, Haifa A. Al-Turki, Mohammed A. AbuJubara

**Affiliations:** a From the Department of Orthopedic Surgery, King Faisal University, Al-Khobar, Saudi Arabia; b From the Department of Internal Medicine, King Fahd Hospital of The University, Al-Khobar, Saudi Arabia; c From the Department of Pharmacy, King Fahd University Hospital, Al-Khobar, Saudi Arabia; d From the Department of Obstetrics and Gynecology, King Fahd University Hospital, Al-Khobar, Saudi Arabia

## Abstract

**BACKGROUND AND OBJECTIVES:**

Glucocorticoid-induced osteoporosis (GIOP) is the most common form of secondary osteoporosis, yet few patients receive proper measures to prevent its development. We retrospectively searched prescription records to determine if patients receiving oral prednisolone were receiving prophylaxis or treatment for osteopenia and osteoporosis.

**METHODS:**

Patients who were prescribed >7.5 milligrams of prednisolone for 6 months or longer during a 6-month period were identified through the prescription monitoring system. Demographic and clinical data were extracted from the patient records, and dual energy x-ray absorptiometry (DEXA) scans were retrieved, when available. Use of oral calcium, vitamin D and anti-resorptives was recorded.

**RESULTS:**

One hundred males and 65 females were receiving oral prednisolone for a mean (SD) duration of 40.4 (29.9) months in males and 41.2 (36.4) months in females. Twenty-one females (12.7%) and 5 (3%) males had bone mineral density measured by DEXA. Of those, 10 (47.6%) females and 3 (50%) males were osteoporotic and 11(52.4%) females and 2 (40%) males were osteopenic. Calcium and vitamin D were prescribed to the majority of patients (60% to 80%), but none were prescribed antiresorptive/anabolic therapy.

**CONCLUSIONS:**

Patients in this study were neither investigated properly nor treated according to the minimum recommendations for the management of GIOP. Physician awareness about the prevention and treatment of GIOP should be a priority for the local health care system.

Osteoporosis is a major public health problem worldwide, with increased morbidity and mortality due to its complications.^1^ Postmenopausal osteoporosis has attained the status of a major epidemic and most women with it present with a fracture as the first indication of the disease.^2^ In the USA, osteoporosis and the treatment of osteoporosisl related fractures costs are estimated to be in excess of $18 billion a year.^2^–^3^ Bubshait and Sadat-Ali^4^ estimated the yearly cost of treating osteoporosis-related femoral fractures in Saudi Arabia as SAR4.27 billion a year. Proper interventions could reduce this cost tremendously.

Glucocorticoids are considered an important component of therapy for a variety of medical conditions including autoimmune, rheumatic, pulmonary and gastrointestinal disorders. Patients treated with glucocorticoids are at risk for many adverse complications.^5^ One of the most important complications associated with long-term use of glucocorticoids is glucocorticoid-induced osteoporosis (GIOP).^6^,^7^ Prolonged use of glucocorticoids causes osteocyte apoptosis with an increase in bone resorption and a decrease in bone formation at both the local and systemic levels, leading to rapid weakening of bone architecture and an increase in fracture risk.^8^,^9^ Evidence indicates that GIOP is the most common cause of secondary osteoporosis, leading to fractures in 30% to 50% of patients taking chronic glucocorticoids.^10^ However, studies showed that many patients treated with glucocorticoids are not properly evaluated and do not receive prophylaxis or treatment to prevent bone loss.^11^,^12^

Osteoporosis secondary to sickle cell disease^13^ and cancer chemotherapy^14^ were reported among the Saudi Arab population. A review of the medical literature found no studies on GIOP in Saudis. We hypoth-esized that evaluation and management of GIOP at our institution is below the standards. This study was carried out to answer the following questions: 1) To what extent is DEXA scanning performed for patients taking glucocorticoids? 2) What percentage of patients taking long-term glucocorticoids are simultaneously prescribed medications to prevent or treat GIOP?

## METHODS

This retrospective study was approved by the research committee of King Faisal University and was carried out at King Fahd Hospital of the University, Al Khobar. We used the local electronic pharmacy prescription monitoring system to identify patients older than 18 years of age, who were prescribed ≥7.5 mg of prednisolone per day for 6 months or longer during the period 1 July 2007 through 31 December 2007. Patients who were prescribed other glucocorticoids apart from oral prednisolone or who took the steroids intermittently were not included in the study.

Subsequently, each patient chart was reviewed for clinical data including age, sex, dose and duration of glucocorticoids therapy. A mean prednisolone dose was taken for patients receiving the minimum daily dose of ≥7.5 milligrams or more for the 6-month period. Dual-energy x-ray absorptiometry (DEXA) scan imaging reports were reviewed when available and data on BMD, T-score and Z-score were documented. Patients with a T score of <2.5 SD were defined as osteoporotic and those between −1 to −2.5 SD were defined as osteopenic for analysis, as defined per the WHO criteria.^16^ Patients who had a DEXA scan were divided into those younger than the age of 35 years, as they had achieved peak bone mass (PBM), and 35 years of age or older. The patient was considered to be screened if he had a BMD measurement while taking glucocorticoid therapy. The concomitant use of osteoporosis prophylaxis or treatment such as calcium, vitamin D, bisphosphonate, calcitonin, and hormonal therapy were noted.

Data was analyzed using a *t* test to compare means between the non-osteoporotic, osteopenic and osteoporotic patients. All tests were performed using SPSS (Statistical Package for the Social Sciences), version 14.0, Chicago, Iinois^15^ a *P* value of <.05 considered statistically significant.

## RESULTS

During the period 1 July 2007 through 31 December 2007, 516 patients received oral corticosteroid therapy according to the local electronic pharmacy prescription monitoring system. One hundred and sixty-five patients (32.0%) received ≥7.5 mg of oral prednisolone per day for 6 months are longer, including 100 males and 65 female patients with a mean (SD) age and range of 37 (12.7) years (range, 7.5-60 years) for males and 40.8 (15) years (range, 18-62 years) for females (Tables 1, 2). Only 26 patients (15.8%) had BMD measured by DEXA scan, including 21 females (32.2% of females) and 5 males (5.0% of males) (*P*=.001) of whom 10 females and 3 males were osteoporotic and 11 females and 2 males were osteopenic (Table 3). None of the patients who had a DEXA scan during glucocorticoid therapy had normal BMD. Fourteen patients were <35 years and 12 were >35 years. In the former group, 10 (71.5%) were osteoporotic compared to 5 (42%) in the latter group. Calcium supplementation was prescribed for 81 (81.0%) males compared to 40 (61.5%) females (*P*=.05). There was no significant difference in the frequency of prescription of vitamin D supplementation between males and females. None of the 165 patients who received ≥7.5 milligrams of steroids daily for 6 months or longer were receiving anti-resorptive/anabolic drugs as prophylaxis or therapy for osteoporosis.

**Table 1:** Primary diseases in patients (n=165) receiving ≥7.5 mg oral prednisolone daily for 6 months or longer.

Primary disease	Number (%)
Asthma	22 (13.3)
Inflammatory bowel disease	15 (9)
Idiopathic thrombocytopenic purpura	13 (7.9)
Chronic nephritis	16 (9.7)
Neuropathies	5 (3)
Rheumatoid arthritis	45 (27.3)
Renal transplant	33 (20)
Systemic lupus erythematosus	16 (9.8)

**Table 2:** Prednisolone therapy and anti-osteoporotic prevention and treatment in 165 patients receiving daily prednisolone for 6 months or longer.

	Male (n=100)	Female (n=65)	*P*(males vs. females)
Mean (SD) dose and range of prednisolone (mg)	15.9 (12.7) (7.5-60)	21.5 (19.5) (7.5-100)	.05
Mean (SD) duration and range of prednisolone (mo)	40.4 (29.9) (6-108)	41.2 (36.4) (6-121)	.2
Treatment with calcium	81 (81%)	40 (61.5%)	.05
Treatment with vitamin D	71 (71%)	38 (58.5%)	.1
Antiresorptives/anabolics	0	0	

**Table 3:** Clinical characteristics of 26 patients who had bone mineral density measured by DEXA scan.

	Male (n=5)	Female (n=21)
Mean (SD) age (years)	50 (17.4)	36.5 (12.1)
Osteopenia (n)	3	11
Osteoporosis (n)	2	10
Mean (SD) spine BMD (g/cm^2^)	0.707 (0.12)	0.783 (0.16)
T score (spine)	-3.2 (0.96)	-2.2 (0.9)
Z score (spine)	-2.24 (0.69)	-1.81 (0.89)
Mean (SD) hip BMD (g/m^2^)	0.632 (0.13)	0.680 (0.15)
T score (hip)	-2.24 (1.03)	-2.04 (0.65)
Z score (hip)	-1.44 (0.68)	-1.45 (0.78)

## DISCUSSION

Secondary osteoporosis is common but still neglected. It is the cause for osteoporosis in almost two-thirds of males, more than half of pre-menopaual and perimenopausal females, and about one-fifth of post-menopausal females.^16^ Etiologies of secondary osteoporosis are many; however, GIOP is one of the leading causes. GIOP and its risk of fragility fractures are well recognized. The combined effect of higher dose, longer duration and a continuous pattern of glucocorticoid therapy could increase vertebral fracture risk by 17-fold and hip fracture risk by 7-fold.^17^,^18^ For this reason, many international guidelines regarding evaluation and treatment of GIOP have been developed. Those guidelines indicate that the use of more ≥5 milligrams for 3 months or longer warrant a DEXA scan to diagnose boss loss and to start prophylactic anti-resorptive therapy using a bisphosphonate or other drugs to prevent osteoporosis.^19^–^22^

Our study showed that during a 6-month period a total of 516 patients were receiving oral predinisolone and 165 patients (32%) were taking ≥7.5 milligrams of steroid per day for 6 months or longer. According to international guidelines, all patients in the later group should have been screened and received effective prophylaxis or therapy for osteoporosis. Despite the fact that we used a higher dose and longer duration of therapy (≥7.5 milligrams for ≥6 months) than what is recommended in recent guidelines,^19^,^20^ only 26 (15.8%) of our patients were evaluated by DEXA scan and all of them had either osteopenia or osteoporosis. A DEXA scan was ordered for only 5 of 100 men. This indicates that physicians are less concerned about the development of osteoporosis in male patients despite the fact that men older than the age of 50 years were found by our group to have osteoporosis of the hip in 24.3% and osteoporosis of the spine in 37.4% in a previous study.^23^ The majority of our patients were taking calcium and vitamin D supplements, which is a higher rate than in other studies,^24^ but unfortunately, none of our patients, including those who were documented to have low bone mass, were prescribed bisphosphonates or any other anti-resorptive or anabolic therapy to prevent or treat osteoporosis. Neglect in providing adequate and recommended prophylaxis or therapy for patients on long-term glucocorticoids appears to be universal. In the study of Gudbjornsson et al (2002),^12^ only 9% of patients were taking a bisphosphonate.

Hart and Green (2002)^25^ found that 64.7% of their patients did not receive the proper prophylaxis for GIOP. Ungprasert et al (2007)^26^ believed that neglect by internal medicine physicians is quite common even in teaching institutions, as in their hospital only 5.8% of patients were evaluated for GIOP while Guzman-Clark and associates (2007)^24^ found that the common barrier for GIOP management was the physician lack of awareness and knowledge.

The mean age of our patients was lower than that reported in other studies.^12^,^23^ This could have been due to different disease patterns and different indications for glucocorticoid therapy. This might also explain the difference in the mean glucocorticoid dose between men and women. In this study we did not evaluate the reasons for steroid therapy or the specialty of the prescribing physicians. Another important finding of our study was the higher incidence of low bone mass in patients who are younger than 35 years of age, which may indicate that patients at a younger age who have not attained PBM are at greater risk of developing GIOP.

Our study has several shortcomings including all those associated with being retrospective. In addition, we did not have vitamin D levels and bone markers available for analysis. Lastly, we did not evaluate the indications of glucocorticoid therapy, which can be a contributing factor to low bone mass.

In conclusion, this study shows that GIOP is neither diagnosed early nor properly managed at our teaching institution. The fact that none of our patients who were on long-term glucocorticoid therapy had been prescribed anti-resorptive drugs raises a concern about the lack of knowledge of international guidelines regarding GIOP. Awareness programs appear to be urgently needed for the physicians prescribing glucocorticoids in order to prevent GIOP and its complications.
